# Vergleich von Patientenbewertungen auf Online-Portalen untereinander und mit Qualitätsberichten der Krankenhäuser und der Qualitätssicherung mit Routinedaten

**DOI:** 10.1007/s00120-023-02263-6

**Published:** 2024-01-24

**Authors:** Conrad Leitsmann, Loraine Kahlmeier, Paul-Oliver Lampe, Christer Groeben, Martin Baunacke, Johannes Huber, Lutz Trojan, Johannes Uhlig, Marianne Leitsmann, Annemarie Uhlig, Arbeitsgruppe „Versorgungsforschung & Digital health“ der GeSRU Academics, Arbeitskreis „Versorgungsforschung, Qualität und Ökonomie“ der DGU e. V.

**Affiliations:** 1 Klinik für Urologie, Universitätsmedizin Göttingen, Georg-August-Universität, Robert-Koch-Str 40, 37075 Göttingen, Deutschland; 2 Klinik für Urologie, Medizinische Universität Graz, Graz, Österreich; 3 Klinik für Urologie, Philipps-Universität Marburg, Marburg, Deutschland; 4 Klinik und Poliklinik für Urologie, Carl Gustav-Carus-Universität Dresden, Dresden, Deutschland; 5 Klinik für diagnostische und interventionelle Radiologie, Universitätsmedizin Göttingen, Georg-August-Universität, Göttingen, Deutschland; 6 aQua – Institut für angewandte Qualitätsförderung und Forschung im Gesundheitswesen GmbH, Göttingen, Deutschland

**Keywords:** Krankenhausbewertungsportale, Patientenbewertungen, Qualitätssicherung mit Routinedaten, Radikale Prostatektomie, Entscheidungshilfe, Hospital rating websites, Patient ratings, Hospital quality report data, Radical prostatectomy, Decision support

## Abstract

**Hintergrund:**

Die Patientenperspektive gewinnt neben objektivierbarer Behandlungsqualität zunehmend an Relevanz.

**Ziel der Arbeit:**

Darstellung verfügbarer Krankenhausportale im Hinblick auf Patientenbewertungen (PaBew) und deren Vergleich mit Daten der Qualitätsberichte der Krankenhäuser und der Qualitätssicherung mit Routinedaten (QSR) für urologische Fachabteilungen.

**Methoden:**

Nach einer strukturierten Online-Recherche nach Bewertungsportalen wurden eingeschlossene Portale untereinander verglichen: PaBew der 10 urologischen Fachabteilungen mit den höchsten Eingriffszahlen im Jahr 2021 wurden mittels „generalized estimated equations“ verglichen. Für die radikale Prostatektomie (RPE) wurde ein quantitativer Vergleich von PaBew („klinikbewertungen.de“) und Bewertungen anhand von QSR-Daten durchgeführt.

**Ergebnisse:**

Die Online-Recherche ergab 1845 Treffer, 25 Portale wurden analysiert. Der Vergleich ergab je nach Portal signifikant unterschiedliche PaBew derselben Fachabteilung (jeweils *p* < 0,001). PaBew und QSR-Daten des „AOK-Gesundheitsnavigators“ zeigten keine signifikante Korrelation. Ein interner Vergleich von QSR-Daten und PaBew aus dem AOK-Gesundheitsnavigator zur RPE zeigte eine signifikante negative Korrelation zwischen Gesamtbewertung und ungeplanten Folgeoperationen (r = −0,81) bzw. sonstigen Komplikationen (r = −0,91). Keine signifikante Korrelation zeigte sich mit der Weiterempfehlungsrate durch Patienten.

**Schlussfolgerung:**

Auf Online-Bewertungsportalen von Krankenhäusern besteht erhebliche Heterogenität bezüglich Patientenbewertungen derselben Fachabteilung je nach verwendetem Portal. Zudem scheint anhand der ausgewählten Beispiele weder eine Korrelation von subjektiven und objektiven Bewertungen zwischen verschiedenen Portalen noch innerhalb eines Portals vorzuliegen.

**Zusatzmaterial online:**

Die Online-Version dieses Beitrags (10.1007/s00120-023-02263-6) enthält weitere Tabellen zur *Suchstrategie bei der Suche nach Online-Portalen*.

## Abstract (en)

**Background:**

In addition to the objectifiable treatment quality, patients’ perspectives are gaining relevance.

**Objective:**

This study aimed to characterize available hospital rating websites (HRW) with regards to patient ratings and to compare them with data from hospital quality reports and quality assurance based on routine data (QSR) for urological departments.

**Materials and methods:**

After a structured online search for HRWs, websites were compared based on patient ratings from the 10 urologic departments with the largest intervention rates in 2021 using generalized estimated equations. For radical prostatectomy (RPE), quantitative comparison of patient ratings (klinikbewertungen.de) and QSR-based ratings was performed using Spearman’s rank correlation.

**Results:**

Of 1845 hits, 25 portals were analyzed. The department-wise comparison of HRWs resulted in significantly different patient ratings (*p* < 0.001). Patient ratings (klinikbewertungen.de) and QSR data (AOK-Gesundheitsnavigator) showed no significant correlation. An internal comparison of QSR data and patient ratings from the AOK-Gesundheitsnavigator on RPE showed a significant negative correlation between the overall rating and unplanned reoperations (r = −0.81) or other complications (r = −0.91). There was no significant correlation with the recommendation rate by patients.

**Conclusion:**

Hospital rating websites show considerable heterogeneity regarding patient ratings of the same urology department in different portals. Furthermore, based on the selected examples, there seems to be no correlation between subjective and objective evaluations between different websites or within one website.

Abseits der objektivierbaren Behandlungsquantität und -qualität spielt die „Patientenmeinung“ eine zunehmend wichtige Rolle bei Bewertung und Vergleich von Behandlungsergebnissen und Krankenhausleistungen. Diese wird neben allgemeinen Informationen und Daten zur Behandlungsqualität zunehmend auf Krankenhaussuchportalen abgebildet. Wir stellten uns die Frage, ob Patientenbewertungen mit objektiven Behandlungsergebnissen vergleichbar sind.

## Hintergrund und Fragestellung

Der Bundestagsbeschluss zum Krankenhaustransparenzgesetz stellt die Schaffung eines Online-Portals für Patienten mit Angaben zu Leistungsspektrum und Behandlungsqualität von Krankenhäusern für Mai 2024 in Aussicht [7]. Ähnliche Portale sind jedoch bereits verfügbar: beispielsweise die „Weisse Liste“ oder der „AOK-Gesundheitsnavigator“ ermöglichen eine gezielte Suche nach Krankenhäusern und bilden, neben allgemeinen Informationen, auch die Behandlungsqualität und/oder bestimmte Leistungen ab. In Deutschland können Patienten auf zahlreiche solcher Portale zurückgreifen, welche jedoch Unterschiede im Hinblick auf Vergleichsmöglichkeiten sowie Datenquellen aufweisen [11]. Die Informationen basieren auf den strukturierten Qualitätsberichten der Krankenhäuser (QB) und/oder der Qualitätssicherung mit Routinedaten (QSR; [3, 17]). Die QB, welche neben Angaben zu Diagnose- und Leistungsspektrum auch Behandlungsergebnisse beinhalten, wurden u. a. aufgrund mangelnder Transparenz und fehlenden Vergleichsmöglichkeiten für Patienten kritisiert [1]. Aus urologischer Sicht ist zudem problematisch, dass, abseits von Nierentransplantationen, keine urologischen Leistungsbereiche erfasst werden. „Qualitätsindikatoren“ der QSR quantifizieren Komplikationen (z. B. Transfusionsraten, ungeplante Folgeoperationen) für beispielsweise die radikale Prostatektomie (RPE) oder Prostataoperationen bei benignem Prostatasyndrom [3].

Die „Patientenmeinung“ spielt eine immer wichtigere Rolle bei der Bewertung von Leistung und Wirksamkeit verschiedener Behandlungen. Ergebnisse von Patientenbefragungen und -bewertungen stehen teilweise auch auf Krankenhaussuchportalen zur Verfügung. Der „patients’ experience questionnaire“ (PEQ) erhebt standardisiert die Zufriedenheit mit z. B. der ärztlichen oder pflegerischen Versorgung und der Organisation [6]. Patientenerfahrungen (PRE) sind neben den von Patienten berichteten Ergebnissen der Behandlung (PRO) sowie den objektivierten medizinischen Behandlungsergebnissen insgesamt eine der anerkannten Ergebnismessgrößen, die neben Parametern der Prozess- und Strukturqualität im Rahmen der Qualitätsbewertung gemessen werden. Folglich ist nicht nur die systematische Erfassung von Patientenerfahrungen i. Allg., sondern auch die Kenntnis über die Inhalte von Patientenbewertungen auf Krankenhausportalen wertvoll.

Ziel dieser Arbeit ist daher, verfügbare Krankenhausbewertungsportale insbesondere im Hinblick auf Patientenbewertungen für urologische Kliniken in Deutschland zu evaluieren und zu untersuchen, ob diese mit objektiven Behandlungsergebnissen vergleichbar sind.

## Methoden

### Recherche nach Krankenhausbewertungsportalen

Es erfolgte eine strukturierte Online-Recherche zur Identifikation von Krankenhausbewertungsportalen. Der Einschluss in weitere Analysen richtete sich nach folgenden Kriterien: 1) Online-Portal mit Evaluation urologischer Fachabteilungen in Deutschland, 2) Bewertung urologischer Fachabteilungen anhand von Daten der QB, QSR-Daten und/oder durch Patienten bzw. anderweitigen Daten der Qualitätskontrolle und 3) Bewertung anhand von Skalen (z. B. Noten, Ampel, Punkte/Sterne). Ausgeschlossen wurden Portale, die lediglich Informationen zu Fachabteilungen lieferten, aber keine Bewertungen. Ebenfalls ausgeschlossen wurden veraltete Portale (letzte Aktualisierung > 2 Jahre) und redundante Portale.

Es wurden Suchmaschinen mit dem zum Suchzeitpunkt größten Marktanteil in Europa verwendet: Google, Bing/Yahoo, Yandex, DuckDuckGo und Ecosia. Die Suche erfolgte im Januar 2022 und enthielt und die im Appendix aufgeführten Operatoren. Vor jeder Suche wurden Cashes und Cookies gelöscht sowie jede Form der Personalisierung und eine lokale Suche möglichst unterbunden. Von jeder Suchmaschine wurden die ersten 25 Treffer weiter gescreent. Enthielten die gescreenten Seiten Links zu potenziell den Einschlusskriterien entsprechenden Portalen, wurden diese weiterverfolgt.

### Beschreibung der Bewertungsportale

Für die eingeschlossenen Portale wurden a priori definierte Daten erhoben: die Angaben zum Portalbetreiber, Betten- und Fallzahlen, Anzahl der urologischen Fachabteilungen, Abbildung von Ergebnissen der QB bzw. QSR- oder ähnlichen Daten und vorhandene Patientenbewertungen. Die Patientenbewertungen wurden nach den in Tabelle B im Appendix genannten Kriterien charakterisiert. Ebenso wurde bei der qualitativen Beurteilung der Online-Portale nach Darstellungs- und Informationsqualität verfahren [16]. Die Datenerhebung erfolgte im Mai 2022.

### Statistische Auswertung

Ein Vergleich der Patientenbewertungen der Portale erfolgte für die 10 urologischen Fachabteilungen mit den insgesamt meisten stationären Behandlungsfällen im Jahr 2021 laut Weisser Liste [8]. Patientenbewertungen der verschiedenen Portale wurden untereinander verglichen. Bei Portalen, welche einzelne Patientenbewertungen separat aufführten, wurden Mittelwerte und Standardabweichungen sowie Mediane und Perzentilen (25 und 75 %) ermittelt. Hierzu wurde eine Normierung der jeweils verwendeten Bewertungsskala auf Prozentwerte vorgenommen. Zudem wurden die Bewertungen mittels „generalized estimated equations“ (GEE) verglichen. Waren nur Mittelwerte sämtlicher Bewertungen verfügbar, erfolgte keine weitere statistische Analyse.

Des Weiteren erfolgte der Vergleich von Bewertungen zur RPE anhand von Patientenbewertungen (klinikbewertungen.de, AOK-Gesundheitsnavigator) vs. QSR-Daten (AOK-Gesundheitsnavigator) mittels Rangkorrelationen nach Spearman. Lediglich im Portal „klinikbewertungen.de“ war ein Maß an Patientenbewertungen verfügbar, welches statistische Vergleiche zuließ. Die ausgewerteten Daten wurden generiert, indem sämtliche verfügbaren Freitextbewertungen nach Begriffen wie „Prostataoperation, Prostatektomie, Prostatakrebs, Prostataentfernung“ durchsucht wurden. Im positiven Fall wurde die jeweilige vergebene Sternezahl der Gesamtbewertung erfasst. Für den AOK-Gesundheitsnavigator erfolgte zudem ein interner Vergleich der QSR-Daten und der Patientenbewertungen. Bei den QSR-Daten wurden die Qualitätsindikatoren Bluttransfusionen, ungeplante Folgeoperationen und sonstige Komplikationen sowie die Gesamtbewertung analysiert. Zudem erfolgte ein Vergleich mit der eingriffsunabhängigen Fallzahl der jeweiligen Fachabteilung im Jahr 2020 laut Weisser Liste und mit der stationären Fallzahl für die RPE im Jahr 2020 laut AOK-Gesundheitsnavigator. Für die Patientenbewertungen des AOK-Gesundheitsnavigators wurde die dort aufgeführte Weiterempfehlungsrate verwendet.

## Ergebnisse

**Abb. 1:**
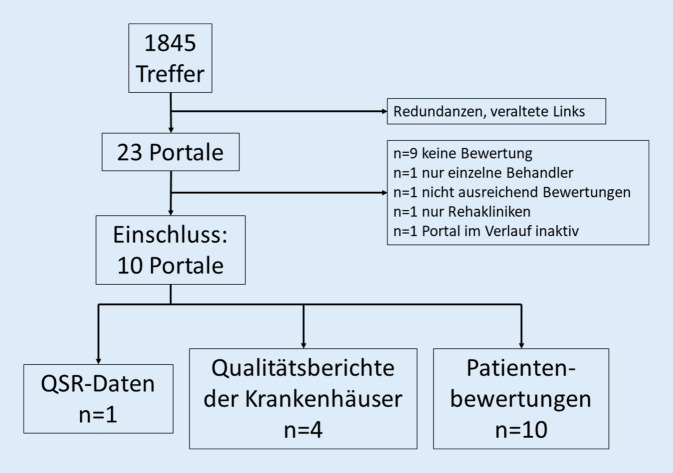
Flussdiagramm für den Einschluss der Portale

Die Online-Recherche ergab 1845 Treffer. Nach Elimination von Redundanzen und veralteten Links wurden *n* = 23 Portale genauer untersucht und *n* = 13 ungeeignete Portale ausgeschlossen (Tab. 1). Von den verbliebenen *n* = 10 eingeschlossenen Portalen veröffentlichten alle Patientenbewertungen anhand von Skalen und *n* = 4 Daten aus den QB sowie *n* = 1 QSR-Daten (Abb. 1; Tabelle C, Appendix).

**Tab. 1:** Krankenhausportale in der finalen Auswahl

Portalname	Betreiber	Link	
*Eingeschlossen*			
AOK-Gesundheitsnavigator	AOK-Bundesverband GbR	https://www.aok.de/pk/cl/uni/medizin-versorgung/krankenhaussuche/	
Google	Google Ireland	https://www.google.de	
Jameda	jameda GmbH	www.jameda.de	
klinikbewertungen.de	MedizInfo® Jürgen Wehner	https://www.klinikbewertungen.de/	
KKH Krankenhaussuche	Weisse Liste gemeinnützige GmbH	https://weisse-liste.krankenhaus.kkh.de/krankenhaus	
krankenhaus.de	Spitality GmbH	https://www.krankenhaus.de/	
krankenhausbewertungen.de	Stephan Brandtstaetter	https://www.krankenhausbewertung.de/	
Sanego	ärzte.de MediService GmbH & Co. KG	https://www.sanego.de	
Weisse Liste	Weisse Liste gemeinnützige GmbH	https://www.weisse-liste.de/	
Yelp	Yelp Ireland Ltd	https://www.yelp.de	
*Ausgeschlossen*			*Grund für Ausschluss*
Deutsches Krankenhaus Verzeichnis	Deutsche Krankenhaus TrustCenter und Informationsverarbeitung GmbH (DKTIG)	https://www.deutsches-krankenhaus-verzeichnis.de/	Keine Patientenbewertung
Arzt-Auskunft	Stiftung Gesundheit	https://www.arzt-auskunft.de/	Nur einzelne Behandler
BARMER Kliniksuche	BARMER	https://www.barmer-kliniksuche.de/	Keine Patientenbewertung
Cylex	S.C Cylex Tehnologia Informatiei International S.N.C.	https://web2.cylex.de/	Nicht ausreichend Bewertungen
DAK Gesundheit Klinikfinder	DAK-Gesundheit	https://www.dak-klinikfinder.de/	Keine Patientenbewertung
der Privatpatient	PKV Verband der Privaten Krankenversicherung e. V.	https://www.derprivatpatient.de/krankenhaus/krankenhaussuche	Keine Patientenbewertung
BKK Klinikfinder	BKK Dachverband e. V.	https://klinikfinder.bkk-dachverband.de/	Keine Patientenbewertung
vdek Kliniklotse	Verband der Ersatzkassen e. V. (vdek)	https://www.vdek-kliniklotse.de/	Keine Patientenbewertung
Klinikradar	Innomeda GmbH	https://klinikradar.de/	Keine Patientenbewertung
HKK Krankenhauslotse	Handelskrankenkasse (hkk)	https://www.hkk-krankenhauslotse.de/	Keine Patientenbewertung
HEK Krankenhauslotse	HEK – Hanseatische Krankenkasse	https://klinikfinder.hek.de/	Keine Patientenbewertung
Hospitalcheck24	Hospitalcheck UG	http://www.hospitalcheck24.net/	Portal im Verlauf inaktiv
Qualitätskliniken	4QD – Qualitätskliniken.de GmbH	https://www.qualitaetskliniken.de/	Nur Rehakliniken

*AOK* Allgemeine Ortskrankenkasse, *BKK* Betriebskrankenkasse, *HEK* Hanseatische Krankenkasse, *HKK* Handelskrankenkasse

Von den 10 eingeschlossenen Bewertungsportalen erfasste das Portal „klinikbewertungen.de“ die meisten Fachabteilungen, wobei hier Doppel- oder Falschnennungen wahrscheinlich sind, da zum Zeitpunkt der Erhebung die Zahl urologischer Fachabteilungen in Deutschland *n* = 394 betrug (Tabelle A, Appendix). Lediglich „krankenhaus.de“ und die „Weisse Liste“ wiesen eine Zertifizierung nach HON-Code auf. 6 von 10 Portalen beinhalteten kostenpflichtige Angebote für Kliniken, dabei war bei 7 Portalen die Darstellung durch die Klinik beeinflussbar. Einen potenziellen Interessenkonflikt sahen wir bei „Yelp“ und der „KKH-Krankenhaussuche“, da hier bezahlte Werbeanzeigen möglich waren. Die Vergleichbarkeit von Fachabteilungen verschiedener Krankenhäuser waren beim AOK-Gesundheitsnavigator und der „Weissen Liste“ gegeben. Nur AOK-Gesundheitsnavigator und „klinikbewertungen.de“ ermöglichten einen Vergleich mit dem nationalen Durchschnitt.

### Patientenbewertungen

Hinsichtlich der Qualität der Patientenbewertung (Tabelle B, Appendix) zeigt sich, dass bei 5 von 10 Portalen ein Account zur Bewertungsabgabe erforderlich war und bei 8 von 10 Portalen die Angabe einer E‑Mail-Adresse. Nähere Angaben waren voraussetzend bei 5 Portalen gefordert. Die Bewertung konnte bei 7 von 10 Portalen anonym abgegeben werden. Das Löschen der Bewertung war bei 4 (Patient) bzw. 5 (Klinik) Portalen möglich, die Bewertungen waren bei 9 Portalen datiert. Bei 8 von 10 Portalen war die Anzahl der eingeflossenen Bewertungen ersichtlich. Transparenz und statistische Auswertung konnten jeweils 3 und 10 Portalen zugeordnet werden. Bis auf „krankenhaus.de“ boten alle 10 Portale Freitextbewertungen an. Ebenso verfügten alle bis auf „KKH-Krankenhaussuche“ über ein skalares Bewertungssystem. Hier zeigten sich allerdings die Skalen wie auch das Labeling (z. B. Noten, Sterne, Herzen) heterogen. Auch die Auswahl der Dimensionen war sehr unterschiedlich. Neben einer einfachen Gesamtwertung konnten bei 5 Portalen mehrere Dimensionen wie z. B. Ausstattung und Kommunikation bewertet werden. Nur der AOK-Gesundheitsnavigator und die Weisse Liste nutzten einen validierten Fragebogen (PEQ). 7 Portale erlaubten den Kliniken, die Bewertungen zu kommentieren, vereinzelt waren Kommentare durch Nutzer oder „Likes“ möglich. Bewertungen anderer Portale bildeten nur 2 Websites ab. In beiden Fällen war jedoch kein Vergleich von Durchschnittsergebnissen möglich. Lediglich bei 2 Portalen wurde das behandelte Krankheitsbild bzw. die durchgeführte Operation zwingend erfasst. Freiwillige Angaben waren bei weiteren 2 Portalen möglich. Teilweise wurden weitere Angaben wie Alter, Geschlecht oder Versicherungsstatus der Patienten erfasst.

### Vergleich von urologischen Fachabteilungen anhand von Patientenbewertungen

Die Tab. 2 zeigt die Mittelwerte und Mediane der auf eine Prozentskala normierten Bewertungen einzelner Portale für die 10 Kliniken mit den höchsten stationären Fallzahlen. Insgesamt waren *n* = 3477 Einzelbewertungen verfügbar. Bereits bei der Anzahl der Bewertungen zeigt sich eine große Spannbreite. Es ist zu beachten, dass nicht für alle Kliniken auf allen Portalen Bewertungen vorlagen. Zudem waren auf der „Weissen Liste“ wie auch bei „Sanego“ keine einzelnen Bewertungen, sondern nur Mittelwerte verfügbar, weshalb keine Streubreiten ermittelt werde konnten. Die 3 Portale mit den meisten Bewertungen waren die „Weisse Liste“, gefolgt von „klinikbewertungen.de“ und „Sanego“.

**Tab. 2:** Mittelwerte und mediane der Bewertungen in einzelnen Portalen für die 10 urologischen Fachabteilungen mit den meisten stationären Fällen im Jahr 2021 laut Weisser Liste [7]

Portal	Mittelwert	Standardabweichung	Median	25 %-Quartile	75 %-Quartile	Anzahl Bewertungen *n* = 1.064
*klinikbewertungen.de*	80,01	29,13	93,33	80,00	100,00	498
*Google*	67,47	43,28	100,00	0,00	100,00	249
*Jameda*	90,79	20,96	100,00	96,00	100,00	285
*krankenhaus.de*	55,56	49,69	100,00	0,00	100,00	9
*krankenhausbewertung.de*	71,48	30,22	83,67	49,67	92,50	22
*Weisse Liste*^*a*^	54,33	NA	NA	NA	NA	1.929
*Sanego*^*a*^	96,67	NA	NA	NA	NA	485

*NA* keine Angabe

^a^Nur Durchschnittswerte je Krankenhaus vorliegend

**Abb. 2:**
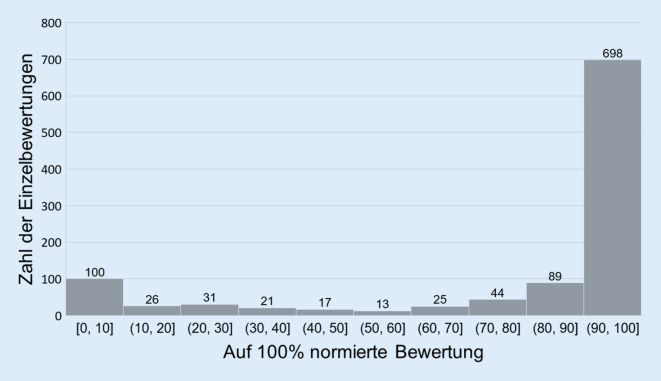
Verteilung der normierten Einzelbewertungen zusammengefasst für die 5 Portale mit verfügbaren Einzelbewertungen („klinikbewertungen.de“, „Google“, „Jameda“, „krankenhaus.de“, „krankenhausbewertung.de“). *Eckige Klammern*: inklusive, *runde Klammern*: >

Die 5 Portale mit *n* = 1063 verfügbaren Einzelbewertungen („klinikbewertungen.de“, „Google“, „Jameda“, „krankenhaus.de“, „krankenhausbewertung.de“) zeigten bei den Bewertungen eine deutlich rechts verzerrte Verteilung, wobei sich das Bewertungsverhalten der Nutzer tendenziell binär gestaltete: die meisten Fälle (66 %) lagen innerhalb der 10. Perzentile, 9 % der Fälle in der 6. Perzentile (Abb. 2).

Der statistische Vergleich der Patientenbewertungen für die 10 Fachabteilungen mit den meisten stationären Behandlungsfällen und individuellen Patientenbewertungen mittels GEE erfolgte für die 5 Portale, in denen individuelle Bewertungen vorhanden waren. Der Vergleich ergab jeweils signifikant unterschiedliche Bewertungen derselben Fachabteilung je Portal (jeweils *p* < 0,001). Patientenbewertungen von „Klinikbewertungen.de“ und QSR-Daten des „AOK-Gesundheitsnavigators“ zeigten keine statistisch signifikante Korrelation (*p* jeweils mindestens ≥ 0,29). Ebenso bestand keine statistisch signifikante Korrelation zwischen Patientenbewertungen von „Klinikbewertungen.de“ und Weiterempfehlungsrate durch die Patienten auf dem AOK-Gesundheitsnavigator (ρ = 0,29, *p* = 0,16).

Ein interner Vergleich von QSR-Daten und Patientenbewertungen des AOK-Gesundheitsnavigators zeigte eine signifikante negative Korrelation zwischen Gesamtbewertung und ungeplanten Folgeoperationen (ρ = −0,81, *p* = 0,02) bzw. sonstigen Komplikationen (ρ = −0,91, *p* < 0,001).

## Diskussion

Patienten in Deutschland haben die Möglichkeit, das Krankenhaus für ihre Behandlung frei zu wählen [2]. Das Verständnis um die Prozesse und Einflussfaktoren für diese Entscheidungsfindung ist daher ein wichtiger Faktor, nicht nur im Wettbewerb der Kliniken [5]. Deutsche Patienten lassen sich hauptsächlich von eigenen Erfahrungen beeinflussen, weitere Informationsquellen sind Angehörige und Behandler sowie in 9,1 % das Internet [4]. In einer kürzlich publizierten Untersuchung zur Krankenhauswahl vor großen uroonkologischen Operationen wie der RPE zeigte sich, dass nur ca. 4,3 % von insgesamt 812 Befragten ein Online-Bewertungsportal zur Entscheidungsfindung verwendeten [9]. Die zunehmende digitale Kompetenz urologischer Patienten sowie eine Verschärfung kompetitiver Verhältnisse im Gesundheitssystem wird die Bedeutung web-basierter Angebote zur Krankenhauswahl weiter in den Fokus rücken. Hinzu kommt, dass nach Groeben et al. fast 30 % der Nutzer eines Bewertungsportals eine Änderung der Entscheidung für ein Krankenhaus durch das Aufsuchen eines Bewertungsportals angaben [9].

Unsere Arbeit hat spezifisch für urologische Kliniken in Deutschland Patientenbewertungen auf verfügbaren Krankenhausbewertungsportalen betrachtet und diese mit objektiven Behandlungsergebnissen verglichen.

### Heterogenität auf Klinikebene

Es besteht eine erhebliche Heterogenität bezüglich der Bewertung derselben Fachabteilung durch verschiedene Online-Portale. Eine Analyse aus dem Jahr 2019 offenbarte bereits eine kaum vergleichbare Datenpräsentation urologischer Inhalte [16]. Bei 70 % der untersuchten Portale war die Darstellung durch die Klinik beeinflussbar. Die objektivste Erfassung der Behandlungsqualität basierend auf Qualitätsindikatoren lieferten erneut der AOK-Gesundheitsnavigator („zuvor AOK-Krankenhausnavigator“) und die „Weisse Liste“.

Auch der Vergleich der Patientenbewertungen ergab signifikant unterschiedliche Bewertungen derselben Fachabteilung auf verschiedenen Portalen (jeweils *p* < 0,001). Die aktuell analysierten 10 Patientenbewertungen für urologische Fachabteilungen sind trotz ihrer Relevanz leider zudem von fraglicher Objektivität, da in der Regel keine validierten Fragebögen genutzt werden bzw. der beim AOK-Gesundheitsnavigator präsentierte PEQ nur Patienten der Krankenkassen AOK oder BARMER einschließt [6].

### Das Potenzial von Patientenbewertungen

In einer Untersuchung amerikanischer Versicherter (Medicare, 2010 und 2011), welche sich einer großen onkologischen Operation unterzogen hatten, zeigten Umfrageergebnisse der American Hospital Association, der Hospital Consumer Assessment of Healthcare Providers and Systems und der Hospital Compare Daten besonders hohe Patientenbewertungen in Verbindung mit höherer Leistung bei Prozessmaßnahmen sowie geringeren Wiederaufnahme- und Mortalitätsraten [15]. Abseits davon spielen für Patienten die Erfahrungen anderer Patienten eine wichtige Rolle. Kuklinski et al. untersuchten Faktoren, die Patienten bei der Wahl des Krankenhauses für eine kolorektale Resektion oder einen Knieersatz beeinflussten. Obwohl auch strukturelle Qualitätsindikatoren eine signifikante Rolle spielten, hatten hohe Patientenbewertungen und Grad der Spezialisierung (z. B. Zertifizierung) den stärksten positiven Einfluss auf die Auswahl [12].

Standardisiert erhobene Patientenergebnisse und -erfahrungen (sog. „patient reported outcome/experience measures“, PROM/PREM) sind bereits fester Bestandteil klinischer Studien. Die Etablierung einer systematischen Erhebung der Patienteneinschätzung zur medizinischen Versorgung ergänzend zu den klinischen Ergebnissen wird zunehmend auch in der Routineversorgung gefordert und sollte vermehrt Platz auf Bewertungsportalen (beispielsweise dem AOK-Gesundheitsnavigator) finden [14]. Gerade am Beispiel der RPE ist neben dem onkologischen Ergebnis die Erfassung funktioneller Ergebnisse wie Harninkontinenz und Impotenz anhand von validierten PROM grundlegend für das operative Ergebnis. Eine Erhebung ist ohnehin im Rahmen von Zertifizierungen gefordert und kann durch eine transparente Darstellung die Beachtung und das Verständnis für solche Ergebnisse verbessern.

In unserer Arbeit korrelierten innerhalb des AOK-Gesundheitsnavigators PEQ-Patientenbewertungen zur RPE allerdings nicht signifikant mit den QSR-Daten. Auch zeigte sich keine Korrelation zwischen AOK-Gesundheitsnavigator Patientenbewertungen und jenen von „klinikbewertungen.de“.

Zukünftige Maßnahmen sollten sich daher auf einen sinnvollen Einsatz von PROM und PREM in Kombination mit etablierten Qualitätsindikatoren fokussieren, um eine standardisierte Auswertung und transparente Darstellung zu ermöglichen.

### Limitationen

Die Bewertung von Online-Quellen ist limitiert durch die Komplexität und Schnelllebigkeit des Internets. Beispielsweise bildet der AOK-Gesundheitsnavigator seit dem letzten Vergleich 2019 die Sterblichkeit innerhalb von 30 Tagen nicht mehr ab [16]. Bezüglich der Vergleiche anhand von QSR-Daten ist der Fokus auf eine einzige Operation sicherlich eine weitere Limitation unserer Arbeit. Urologische QSR-Daten werden aktuell jedoch nur für die RPE abgebildet. Um eine portalübergreifende statistische Vergleichbarkeit mit Patientenbewertungen zu ermöglichen, wurden zudem die 10 Kliniken mit den insgesamt meisten stationären Fällen verwendet und nicht jene Kliniken mit den meisten RPE-Eingriffen. Unsere aktuelle Arbeit beinhaltet keine Untersuchung der Bewertung der Behandlung durch niedergelassene Urologen. Dies wäre ein gezielter Ansatz für eine weitere Untersuchung.

Beim Vergleich der Patientenbewertungen waren auf 2 der Portale mit den meisten Bewertungen („Weisse Liste“ und „Sanego“) keine einzelnen Bewertungen, sondern nur Mittelwerte verfügbar, weshalb keine Streubreiten ermittelt werden konnten und kein Vergleich mittels GEE erfolgte.

Abschließend sei bemerkt, dass die Normierung von unterschiedlichen Bewertungsskalen auf eine 100 %-Skala und ein abschließender Vergleich mittels GEE keine mathematisch perfekte Lösung darstellt. Gerade Skalen mit nur wenig Spielraum in der Bewertungsvergabe (z. B. 1–3 Sterne) können zu einer Verzerrung von gemittelten Werten und Streuungsmaßen führen und untergraben die Feingranularität von Bewertungen z. B. auf einer 10-Punkte-Skala. Aus diesem Grund haben wir von einem direkten statistischen Vergleich der Mittelwerte in Tab. 2 abgesehen und den Vergleich über ein GEE gewählt, auch wenn dies ebenfalls starke Annahmen bezüglich eines vergleichbaren Bewertungsverhaltens von Patienten unabhängig von Bewertungsskalen enthält.

## Ausblick

Die Wahrnehmung und die Nutzung von Krankenhausbewertungsportale werden in Zukunft zunehmen. Zusätzlich wird 2024 im Rahmen des Krankenhaustransparenzgesetztes ein weiteres Online-Portals geschaffen werden. Die in unserer Arbeit gezeigte erhebliche Heterogenität sowohl zwischen einzelnen Portalen als auch innerhalb der subjektiven und objektiven Bewertungen eines Portals schränkt den Nutzen der Portale für Patienten wesentlich ein. Des Weiteren hinterlassen die Beeinflussbarkeit der Darstellung durch die Kliniken und die Intransparenz der Portale einen unseriösen Beigeschmack. Zukünftige Bestrebungen von Portalbetreibern sollten auf eine Verbesserung der Qualität der dargestellten Inhalte abzielen, da Nutzerorientierung wiederum die Akzeptanz von Online-Angeboten bedingt [10, 13]. Auch standardisierte Erhebungen mittels PREM/PROM sowie die Einbeziehung von medizinischen Fachgesellschaften beinhalten erhebliches Verbesserungspotential.

## Fazit für die Praxis

- Vergleiche von Krankenhausportalen zeigten signifikant unterschiedliche Patientenbewertungen derselben Fachabteilung.
- Eine Korrelation von subjektiven und objektiven Bewertungen liegt weder zwischen verschiedenen Portalen noch innerhalb eines Portals vor.
- Standardisierte Erhebungen mittels PREM/PROM („patient reported outcome/experience measures“) sollten zukünftiger Bestandteil von Erhebungen zur Behandlungsqualität sein.

### Supplementary Information

Suchstrategie bei der Suche nach Onlineportalen; Beschreibung und Qualitätskriterien der 10 eingeschlossenen Krankenhausbewertungsportale; Beschreibung und Inhalte von Patientenbewertungen der 10 eingeschlossenen Krankenhausbewertungsportale; Ergebnisse AOK-Gesundheitsnavigator für die 10 Urologischen Kliniken mit den meisten stationären Fällen im Jahr 2021.
