# Supplementary material for: Comparison of hospital rating websites among each other and with data from hospital quality reports and quality assurance based on routine data
Source: Urologie. 2024 Jan 24;63(5):474–81. [Article in German] doi: 10.1007/s00120-023-02263-6 (PMC11090928; doi:10.1007/s00120-023-02263-6)
Supplement: Supplementary file 1 — Suchstrategie bei der Suche nach Onlineportalen; Beschreibung und Qualitätskriterien der 10 eingeschlossenen Krankenhausbewertungsportale; Beschreibung und Inhalte von Patientenbewertungen der 10 eingeschlossenen Krankenhausbewertungsportale; Ergebnisse AOK-Gesundheitsnavigator für die 10 Urologischen Kliniken mit den meisten stationären Fällen im Jahr 2021. [file 120_2023_2263_MOESM1_ESM.docx]

**Appendix**

Suchstrategie bei der Suche nach Onlineportalen

„Krankenhausbewertung OR Krankenhaus AND Bewertungsportal OR Krankenhaus AND Bewertung OR Klinikbewertung OR Krankenhausempfehlung OR Krankenhaus AND Empfehlung OR Klinikempfehlung OR Klinik AND Empfehlung OR Krankenhaussuche OR Patientenbewertung AND Krankenhaus“

Tabelle A. Beschreibung und Qualitätskriterien der 10 eingeschlossenen Krankenhausbewertungsportale

| **Portal** | AOK-Gesundheit-snavigator | Google | Jameda | klinikbewertungen.de | KKH Krankenhaussuche | krankenhaus.de | krankenhausbewertung.de | Sanego | Weisse Liste | Yelp |
| --- | --- | --- | --- | --- | --- | --- | --- | --- | --- | --- |
| Betreiber | AOK-Bundesverband GbR | Google Ireland | jameda GmbH | MedizInfo® Jürgen Wehner | Weisse Liste gemeinnützige GmbH | Spitality GmbH | Stephan Brandtstaetter | ärzte.de MediService GmbH & Co. KG | Weisse Liste gemeinnützige GmbH | Yelp Ireland Ltd. |
| Angabe von Betten- und Fallzahl | + | - | - | - | - | + | + | - | + | - |
| Evaluation der Behandlungsqualität spezifisch für urologische Fachabteilungen/Krankheitsbilder/Eingriffe | + | - | - | - | + | + | - | - | + | - |
| Qualitäts-berichte der Krankenhäuser | + | - | - | - | + | ~ | + | - | + | - |
| QSR-Daten | + | - | - | - | - | - | - | - | - | - |
| Patientenbewertungen | + | + | + | + | + | + | + | + | + | + |
| Zahl der urologischen Fachabteilungen | 479 | ~ | ~ | 650 | 394 | 496 | 516 | 27 | 394 | ~ |
| Werbung | - | + | - | + | - | + | - | + | - | + |
| Zertifizierung nach HON-Code | - | - | - | - | - | + | - | - | + | - |
| Kostenpflichtige Angebote für Patienten | - | - | - | - | - | - | - | - | - | - |
| Kostenpflichtige Angebote Kliniken | - | - | + | + | - | + | - | + | - | + |
| Beinflussbare Darstellung durch Klinik | + | - | + | + | + | + | + | + | - | + |
| Potenzieller wirtschaftlicher Interessenkonflikt Websitebetreiber | - | - | - | - | +* | - | - | - | - | +* |
| Vergleichbarkeit (urologischer) Fachabteilungen verschiedener Krankenhäuser | + | - | - | - | + | - | - | - | + | - |
| Vergleichbarkeit (urologischer) Fachabteilungen mit dem nationalen Durchschnitt | + | - | - | + | + | - | - | - | - | - |
| Pop-up Buttons/ Links zur Erklärung | + | + | + | - | + | - | - | - | + | - |
| Filterfunktionen | + | - | - | - | + | teilweise | - | - | teilweise | + |
| Aktualität und Funktionalität von Links | + | + | + | + | + | + | + | teilweise | + | + |

*Legende: +=ja, -=nein, ~=unklar; QSR=Qualitätssicherung mit Routinedaten, HOM=Health on the internet, KKH=Kaufmännische Krankenkasse; * Grund: bezahlte Werbeanzeigen und entsprechend bessere Sichtbarkeit*

Tabelle B: Beschreibung und Inhalte von Patientenbewertungen der 10 eingeschlossenen Krankenhausbewertungsportale.

| **Portal** | AOK-Gesundheitsnavigator | Google | Jameda | klinikbewertungen.de | KKH Krankenhaussuche | krankenhaus.de | krankenhausbewertung.de | Sanego | Weisse Liste | Yelp |
| --- | --- | --- | --- | --- | --- | --- | --- | --- | --- | --- |
| Account zur Bewertungsabgabe erforderlich | - | + | + | + | - | - | - | + | - | + |
| Angabe Emailadresse erforderlich | - | + | + | + | + | + | - | + | + | + |
| Weitere Notwendige Angaben | Geschlecht, Geburtsjahr, Bildungsgrad | keine | freiwillig: Grund und Dauer Aufenthalt, Ver-sicherungsstatus | Privatperson oder Klinikmitarbeiter | Patient/Angehörige/ Klinikmitarbeiter | keine | Alter, Versicherungsstatus, Diagnose | freiwillig: Versicherungsstatus, Alter, Geschlecht | Geschlecht, Geburtsjahr, Bildungsgrad | keine |
| Bewertungen anonym | + | - | + | + | + | - | + | + | + | - |
| Löschen der Bewertung durch Patienten bzw. Klinik möglich? | - | + | + | - | ~ | ~ | + (Patient: teilweise) | - | Patient: -  Klinik: + | + |
| Datierung von Bewertungen | - | + | + | + | + | + | + | + | + | + |
| Anzahl der eingeflossenen Bewertungen ersichtlich | teilweise | + | + | + | + | - | + | + | + | + |
| Transparenz statistische Auswertung | + | teilweise | - | - | - | - | - | - | - | teilweise |
| Freitext | + | + | + | + | + | - | + | + | + | + |
| Bewertungssystem | Noten: 1-6 | Symbol: 1-5 | Noten: 1-6 | Symbol: 0-6, Punkte: 1-4 | - | Symbol: 1-5 | Symbol: 0-5, Noten: 1-6 | Punkte: 1-10 | Noten: 1-6 | Symbol: 1-5 |
| Bewertungskriterien | PEQ | - | medizinische Versorgung, Aufklärung, Vertrauensverhältnis, Freundlichkeit Wartezeit, Mahlzeiten, Besuchszeiten, Ausstattung Hygiene, öffentliche Erreichbarkeit | Gesamt-zufriedenheit, Beratung, medizinische Versorgung, Verwaltung und Abläufe, Ausstattung | - | - | medizinische Versorgung, Aufnahme, Wartezeiten, Kommunikation, Serviceleistungen, Hygiene, Ausstattung, Mahlzeiten | Behandlungserfolg, ärztl. Kompetenz,  Beratung durch den Arzt, Kommunikation, Terminvereinbarung Wartezeit, Freundlichkeit, Ausstattung, Hygiene, Weiterempfehlung | Ärztl. Behandlung, Kommunikation, pflegerische Betreuung, Wartezeit, Aufnahme, Sauberkeit, Mahlzeiten, Entlassung, Weiterempfehlung | - |
| Kommentar von Bewertungen möglich | - | +, Klinik und Likes durch Nutzer | +, Klinik | + | +, Klinik | - | +, Klinik | +, Klinik und Likes durch Nutzer | - | +, Klinik |
| Abbildung von Bewertungen anderer Portale | - | + | - | - | - | +, Google | - | - | - | *~* |
| Erfassung von Krankheitsbildern und/oder Operationen | - | - | freiwillig | freiwillig | + | - | + | - | - | - |
| Weitere Inhalte | - | - | Zeit-punkt der Behandlung, Dauer des Aufenthaltes, Versicherungsstatus, Weiterempfehlung | Jahr der Behandlung, Versicherungsstatus, Weiterempfehlung | - | - | Weiterempfehlung, Alter, Versicherungsstatus | Zeitpunkt der Behandlung, verschriebene Medikamente, Versicherungsstatus, Alter, Geschlecht | Aktueller Gesundheitszustand, Alter, Geschlecht, Bildungsgrad | - |

*Legende: +=ja, -=nein, ~=unklar; QSR=Qualitätssicherung mit Routinedaten, KKH=Kaufmännische Krankenkasse, PEQ=* *Patients' Experience Questionnaire; *Sterne/Haken/Herzen*

Tabelle C. Ergebnisse AOK-Gesundheitsnavigator für die 10 Urologischen Kliniken mit den meisten stationären Fällen im Jahr 2021.

| Rangwertung nach Fallzahl | Stationäre Fallzahl lt. Weisser Liste 2020 | Universitätsklinikum | Eingriffszahl RPE lt. AOK 2020 | Fallzahl AOK  2018-2020 | Gesamtbewertung  SMR (95%KI)* | Bluttransfusion innerhalb von 30 Tagen  SMR (95%KI)* | Ungeplante Folge-Operation innerhalb von 365 Tagen  SMR (95%KI)* | Sonstige Komplikationen innerhalb von 30 Tagen  SMR (95%KI)* | Weiterempfehlung lt. AOK [%] |
| --- | --- | --- | --- | --- | --- | --- | --- | --- | --- |
| **1** | 5454 | nein | 108 | 114 | 0.7 (0.3-1.1) | 0.3 (0.0-1.4) | 0.8 (0.2-1.4) | 0.8 (0.0-1.5) | 82 |
| **2** | 4874 | nein | 486 | 497 | 1.3 (1.1-1.5) | 2.5 (2.1-3.0) | 0.8 (0.5-1.1) | 1.2 (0.9-1.6) | 87 |
| **3** | 4789 | ja | 624 | 191 | 1.0 (0.6-1.4) | 0.8 (0.0-1.8) | 1.2 (0.7-1.7) | 0.8 (0.0-1.5) | 87 |
| **4** | 4586 | ja | 355 | 118 | 1.6 (1.2-2.0) | 1.0 (0.1-1.9) | 1.8 (1.2-2.4) | 1.7 (1.1-2.3) | 80 |
| **5** | 4087 | ja | 532 | 243 | 0.8 (0.5-1.1) | 0.4 (0.0-1.2) | 0.9 (0.4-1.3) | 0.8 (0.2-1.3) | NA |
| **6** | 4085 | ja | 132 | 74 | 0.6 (0.0-1.1) | 1.3 (0.1-2.6) | 0.5 (0.0-1.3) | 0.2 (0.0-1.2) | 80 |
| **7** | 3983 | nein | 76 | 63 | 0.8 (0.4-1.3) | 1.0 (0.1-1.8) | 1.0 (0.2-1.7) | 0.5 (0.0-1.3) | 80 |
| **8** | 3851 | nein | 40 | NA | NA | NA | NA | NA | NA |
| **9** | 3849 | nein | 37 | NA | NA | NA | NA | NA | NA |
| **10** | 3814 | nein | 360 | 152 | 0.3 (0.0-0.7) | 0.2 (0.0-1.2) | 0.3 (0.0-0.9) | 0.1 (0.0-0.8) | 73 |

*Legende: AOK=Allgemeine Ortskrankenkasse, KI=Konfidenzintervall, NA=keine Angabe, SMR= Standardisierte Mortalitäts-Ratio*
